# Toxicity of graphene oxide to naked oats (*Avena sativa* L.) in hydroponic and soil cultures†

**DOI:** 10.1039/c8ra01753k

**Published:** 2018-04-24

**Authors:** Lingyun Chen, Shengnan Yang, Ying Liu, Min Mo, Xin Guan, Liu Huang, Chao Sun, Sheng-Tao Yang, Xue-Ling Chang

**Affiliations:** College of Chemistry and Environment Protection Engineering, Southwest Minzu University Chengdu 610041 P. R. China yangst@pku.edu.cn; CAS Key Laboratory for Biomedical Effects of Nanomaterials and Nanosafety, Institute of High Energy Physics, Chinese Academy of Sciences Beijing 100049 P. R. China changxl@ihep.ac.cn

## Abstract

Graphene nanomaterials are emerging environmental pollutants and their toxicity to plants requires careful investigation in environmental matrixes. Actually, the transportation of graphene in hydroponic systems is completely different to that in soil, which might affect the interaction between graphene and plants. In this study, we compared the toxicity of graphene oxide (GO) to naked oats (*Avena sativa* L.) in hydroponic and soil cultures. Serious toxicity of GO was only observed in hydroponic culture. GO induced the inhibition of biomass gain, seedling length and photosynthesis of naked oats. The root structure was disturbed by GO and oxidative stress was aroused in the root. In contrast, the soil (vermiculite) interacted strongly with GO and restricted the transportation of GO in soil. This reduced the contact between GO and the roots and largely alleviated its toxicity. Our results collectively suggested that environmental biosafety evaluation should consider the impact of environmental behaviors of nanomaterials to better reflect the real bioeffect of nanomaterials.

## Abstract 2

Graphene oxide showed much higher toxicity to plants in hydroponic culture than in soil culture.

## Graphical abstract

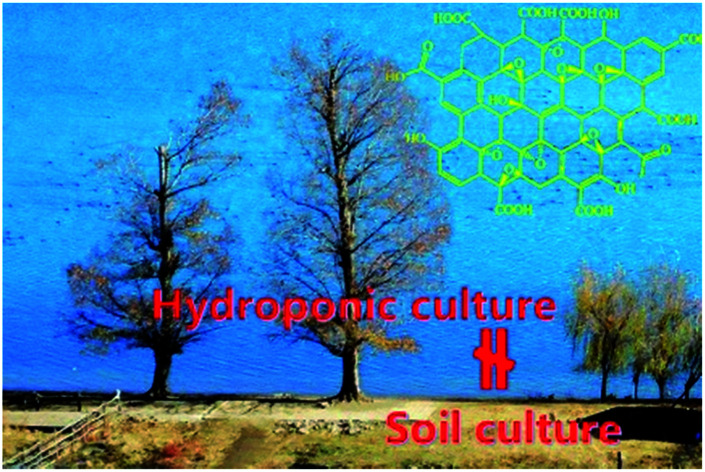

## Introduction

Since its discovery, graphene has had tremendous applications in various areas due to its unique structures and properties.^1–4^ Graphene is a single-layered hexagonal mesh formed by sp^2^ carbon atoms in six-membered rings. During oxidation, some aromatic rings are broken and oxygen containing groups are added onto the carbon atoms, including epoxy, hydroxyl and carboxyl groups, and the graphene is converted into graphene oxide (GO).^5–8^ Despite the damage to the intact structure of graphene, the introduction of oxygen containing groups largely increases the hydrophilicity and water dispersibility of graphene, and allows diverse functionalization reactions on these groups to produce graphene derivatives for applications. GO is now among the most important carbon nanomaterials with applications in the environment,^9–11^ biomedicine,^12^ catalysis,^13^ agriculture,^14^ and so on. Beyond this, due to the easy preparation protocol and low production cost, GO is the most important precursor for producing graphene *via* chemical reduction.^15^ Therefore, there is a growing demand for GO and nowadays production lines are operating with an annual production capacity of several hundred tonnes in China.^16^

The fast growing production and applications of GO have aroused environmental biosafety concerns among the scientific community and also the public.^17,18^ Many studies have reported the bio-effects of GO on diverse models. At the very beginning, Chang *et al.*^19^ reported that GO induced oxidative stress in A549 cells in a dose and size dependent way. GO was also reported to be toxic to bacteria and fungi, leading to proliferation inhibition, structural disorder and function loss.^20^ Toxic effects were observed in animals upon administration *via* different pathways.^21^ Very recently, we found that GO bioaccumulated in plant roots, caused oxidative stress there, and consequently led to serious toxicity in wheat.^22^ Based on the available data in the literature, GO is regarded as a potential pollutant and its environmental risk requires thorough investigation.

Among the environmental safety concerns for GO, the toxicity of GO to plants has recently received a lot of attention. For example, many researchers have explored the effect of GO on plant growth under hydroponics.^23–26^ GO does not influence the germination and development of *Arabidopsis thaliana* at concentrations of 0–1000 μg L^−1^, while severe GO accumulations in root hair and root parenchyma cells were observed under TEM.^23^ Anjum *et al.* found GO induced toxicity in faba beans (*Vicia faba* L.), including growth inhibition and oxidative stress.^24^ Hu *et al.* reported that GO amplified the toxicity of arsenic in wheat under co-exposure to GO and pollutants.^25^ The adverse effects of graphene on the germination and seedling morphology of rice in hydroponic culture were investigated by Liu *et al.*^26^ On the other hand, GO diminished the toxicity of copper to duckweed (*Lemna minor* L.).^27^ Moreover, Chakravarty *et al.*^14^ reported that graphene quantum dots enhance the growth of coriander and garlic plants in soil culture systems, which was quite different to the results for GO in the hydroponic system. Although very few studies have evaluated graphene structure and properties in soil culture systems, these differences may be attributable to the changes of the carbon nanomaterial structure/properties and the strong adhesion of carbon nanomaterials to soil.

It is well known that the morphological and physicochemical properties of GO vary in different media, which would influence its transportation and fate in the environment.^28^ Generally, GO disperses well in water and the diffusion of GO is easy in liquid systems, while the properties of GO are regulated by the liquid properties. For example, Wu *et al.* found that the size, surface chemistry and electrochemical properties of GO changed in water media at different pH values.^29^ Liu *et al.* showed using a sand column that retention and transportation of GO in porous media strongly depended on the solution ionic strength.^30^ GO mobility dramatically reduced with the increase of ionic strength. Similarly, the adhesion of GO to quartz sand became favourable with the increase of NaCl concentration.^31^ Additionally, when GO entered soil, GO–soil binding that restricted the transportation of GO in soil could occur.^31–33^ Many other studies also observed the retention and binding of GO in soil.^32,33^ Thus, when plants are exposed to GO in different media, the transportation of GO would be different and might lead to completely different bio-effects. This is crucial for the toxicity and biosafety of nanomaterials. Thus, during bio-effect evaluation of GO, the interaction between GO and the soil has to be taken into consideration to reflect its real bio-effects.

In this study, we evaluated the toxicity of GO to naked oats (*Avena sativa* L.) that were planted in hydroponic and soil culture systems, respectively. GO was supplemented into vermiculite (a simulation of soil) and Hoagland nutrient solution for plant exposure. The fresh and dry weights, seedling lengths, root numbers, photosynthesis related parameters, histological observations and ultrastructure were investigated to compare the toxicity of GO in the two different culture systems. Oxidative stress was assayed to reveal the potential toxicological mechanism. The transportation and binding of GO in vermiculite were studied to explain the different bio-effects of GO in the two systems. The implication to future environmental hazard assessments of graphene materials is discussed.

## Results and discussion

### Characterization of GO

**Fig. 1:**
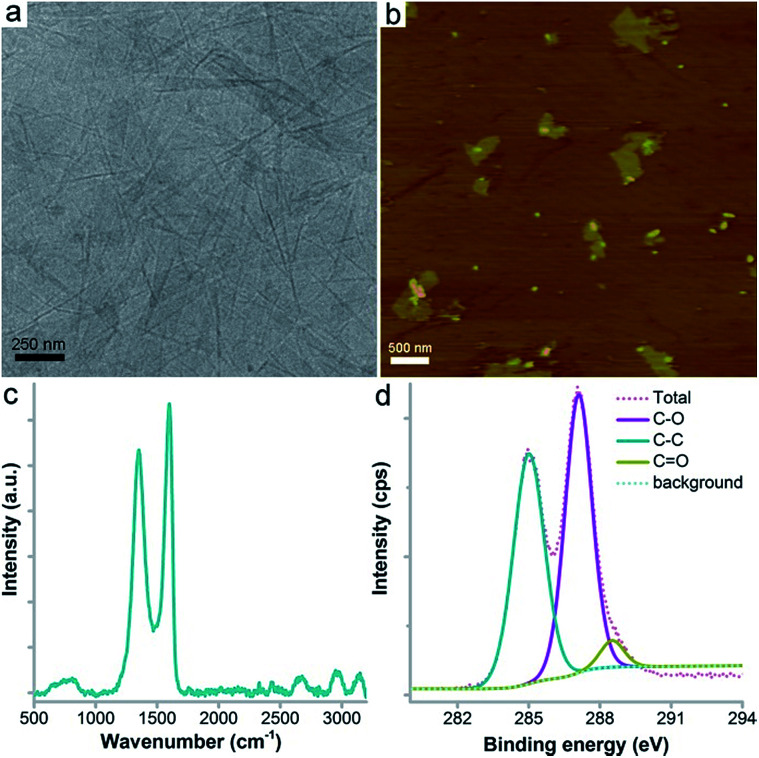
Characterization data for GO. (a) TEM image; (b) AFM image; (c) Raman spectrum; (d) C1s XPS spectrum.

GO was obtained as a brown dispersion upon sonication that showed sheet structures under TEM and AFM (Fig. 1). Under TEM, the folding and stacking of GO sheets were observed, which are due to the drying process for sampling (Fig. 1a). The AFM image showed flat sheets with much less stacking and folding (Fig. 1b). The sheet height was about 1 nm, which is consistent with the literature results. The typical sp^2^ carbon atoms were reflected by the Raman G band signal at 1598 cm^−1^ (Fig. 1c). The oxidation induced defects were indicated by the strong D band signal at 1350 cm^−1^. The weaker 2D and 2G bands suggested the stacking of GO sheets during lyophilisation. The chemical components of GO were investigated using XPS, which showed the existence of C (66.2 at%), O (32.0 at%) and N (1.8 at%) atoms. The C atoms were further divided into three components (Fig. 1d), namely C–C (46.8%), C–O (49.1%) and C=O (4.1%). The abundant oxygen containing groups were confirmed using IR analysis (Fig. S1†). The –OH/–COOH groups were reflected by the peak at 3349 cm^−1^. The peak at 1730 cm^−1^ represented the C=O bonds and the peak at 1225 cm^−1^ indicated the existence of C–O bonds. The peak at 1629 cm^−1^ was assigned to the C=C bonds. The characterization data collectively suggested that the GO sample was of high purity and suitable for the following toxicity evaluation.

### Growth inhibition

**Fig. 2:**
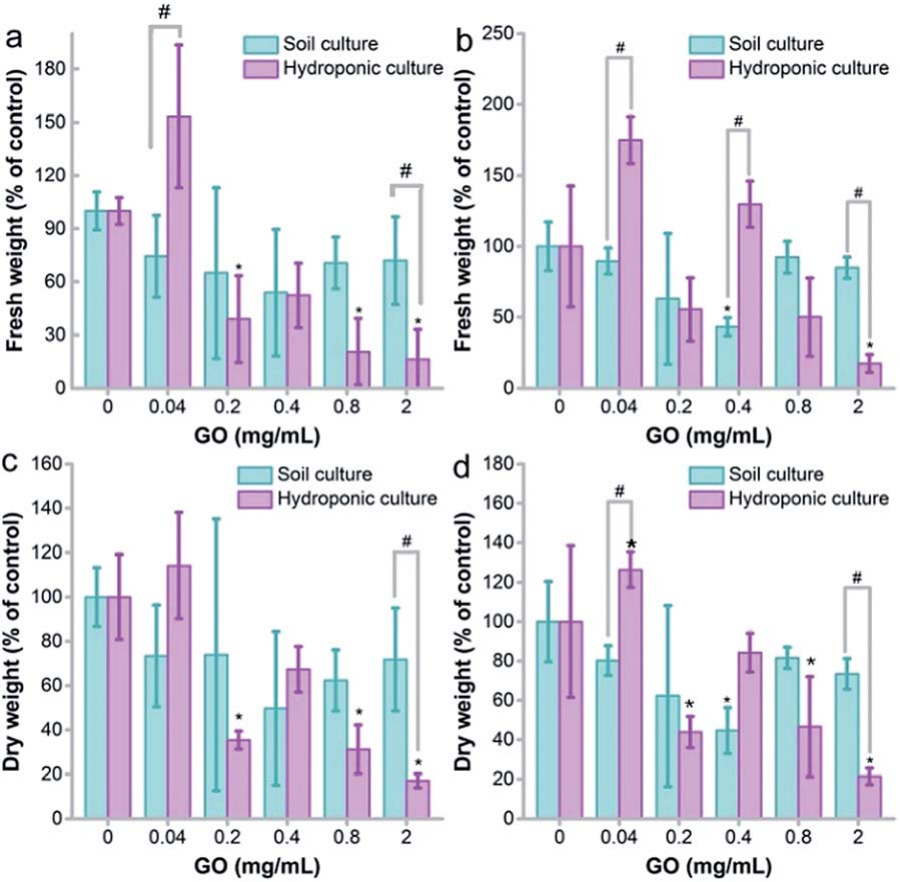
Biomass gain of naked oats exposed to GO in soil culture and hydroponic culture (*n* = 5). (a) Fresh weights of root samples; (b) fresh weights of aboveground parts; (c) dry weights of root samples; (d) dry weights of aboveground parts. * *p* < 0.05 compared to the control group; # *p* < 0.05 between soil and hydroponic culture.

The fresh weight gains of naked oats were slightly inhibited for both soil and hydroponic cultures, but the decrease was more obvious and statistically significant in hydroponic culture (Fig. 2). The root samples showed fresh weight loss at GO concentrations of 0.2 mg mL^−1^ and higher (*p* < 0.05). No statistical difference was observed between the two cultivation modes. The dry weights of the root samples had a significant difference between the two modes at a GO concentration of 2.0 mg mL^−1^, indicating the higher toxicity to roots in hydroponic culture. For leaf samples, the fresh weight increased at 0.04 mg mL^−1^ GO and decreased at 2.0 mg mL^−1^. The stimulation at low concentration and inhibition at high concentration were also observed in wheat and other plant models.^22^ Similar results were obtained for the dry weights.

For soil culture, the only inhibition was observed at a GO concentration of 0.4 mg mL^−1^ for both fresh and dry weights. At 2.0 mg mL^−1^, the toxicity of GO was more serious according to the fresh and dry weights in hydroponic culture (*p* < 0.05). For the dry/fresh ratio, the root showed an increasing trend in hydroponic culture, but a decrease of the dry/fresh ratio was found in soil culture (Fig. S2a†). For the aboveground parts, GO induced an increase of the dry/fresh ratio at 2.0 mg mL^−1^ in hydroponic culture (*p* < 0.05) and the increase was also statistically significant between hydroponic and soil cultures (Fig. S2b†). The increase of the dry/fresh ratio is due to the stimulation of fresh water, which meant that a higher water content was detected rather than a real biomass increase.

**Fig. 3:**
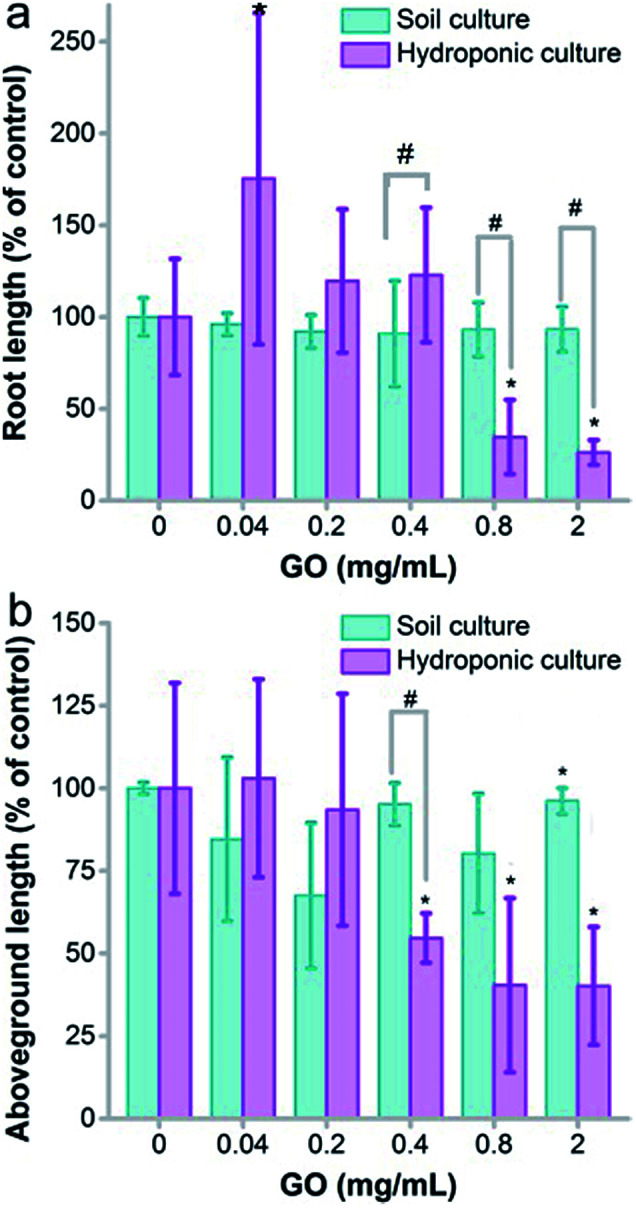
Seedling length of naked oats exposed to GO in soil culture and hydroponic culture (*n* = 5). (a) Root length; (b) aboveground part length. * *p* < 0.05 compared to the control group; # *p* < 0.05 between soil and hydroponic culture.

The growth inhibition was reflected by the seedling lengths, too. As shown in Fig. 3a, the root length of naked oats was not influenced by GO in soil culture. Serious inhibition of root lengths was observed in hydroponic culture at GO concentrations of 0.8 and 2.0 mg mL^−1^. The difference was significant between hydroponic and soil cultures (*p* < 0.05), indicating that GO was more toxic to roots in hydroponic culture. The situation was the same for the aboveground parts. GO inhibited the aboveground part lengths at 0.4 mg mL^−1^ and higher, whereas very slight inhibition was found for soil culture only at 2.0 mg mL^−1^. Again, the difference between the two cultivation modes was significant. When counting the root number and leaf number, the root number decreased at GO concentrations of 0.4–2.0 mg mL^−1^ and the leaf number decreased at 0.8 and 2.0 mg mL^−1^ in hydroponic culture (Fig. S3a and b†). No influence of GO on the root and leaf numbers was observed in soil culture. In addition, the root/shoot ratio showed an increase at low GO concentrations and decreased at high GO concentrations (Fig. S3c†).

The growth inhibition of naked oats by GO was reasonable and consistent with the literature results. Begum reported that GO inhibited the root lengths (78%) and the aboveground part lengths (61%) of cabbage at 2 mg mL^−1^.^34^ Similar inhibition was also observed in the biomass gain of roots (88%) and aboveground parts (81%). Cheng *et al.* observed the inhibition effect of GO on *Brassica napus* L.^35^ The root length decreased by 55% and the root weight decreased by 43% at a GO concentration of 100 mg L^−1^. In our study, inhibition by GO was observed in root lengths (26%) and aboveground part lengths (40%) in soil culture at 2.0 mg mL^−1^, which is much lower than that in hydroponic culture (93% for roots and 96% for aboveground parts). Another interesting phenomenon was the stimulating effect of GO at a low concentration of 0.04 mg mL^−1^ in hydroponic culture. This was attributed to hormesis, which was also reported by other groups. For example, Liu *et al.* observed an increase of the root number and weight of rice at 5 mg L^−1^ graphene.^26^ Zhang *et al.* reported the stimulation of root and leaf elongation by graphene using wheat as the model.^36^

The higher toxicity of GO in hydroponic culture might not be surprising. According to the literature reports, the same nanomaterials have different biological effects in different culture media. For instance, Khodakovskaya *et al.* reported that carbon nanotubes enhanced the growth of tobacco cell culture (55–64% increase over control) at concentrations of 5–500 μg mL^−1^ on a Murashige and Skoog medium with 0.8% agar.^37^ However, Begum *et al.* found that the carbon nanotube exposed plants exhibited growth inhibition and cell death in hydroponic culture.^38^ Zinc oxide nanoparticles are biologically toxic to plants in aqueous suspension culture systems.^39^ However, Wang *et al.* found that the toxicity of zinc oxide nanoparticles to corn was reduced in soil culture systems containing *Arbuscular mycorrhizae*.^40^ In combination with the literature results, we suggest that the culture systems should be carefully considered when evaluating the toxicity of nanomaterials to plants.

### Photosynthesis

**Fig. 4:**
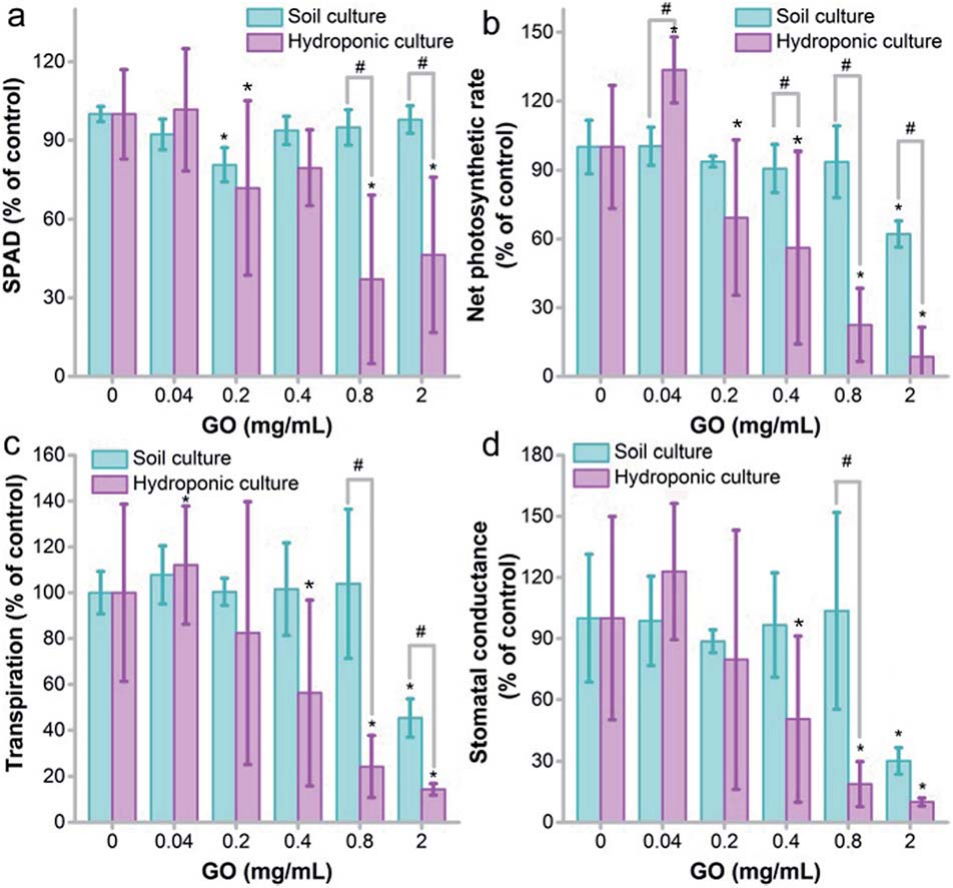
Physiological characteristics of naked oats exposed to GO in soil culture and hydroponic culture (*n* = 5). (a) Chlorophyll content; (b) net photosynthetic rate; (c) transpiration; (d) stomatal conductance. * *p* < 0.05 compared to the control group; # *p* < 0.05 between soil and hydroponic culture.

Photosynthesis is the key function of plants that converts CO_2_ into organic matter, which is vital for the growth of the plant and the carbon cycle. Unlike our previous evaluation on wheat, where GO did not disturb the chlorophyll content,^22^ here we found that GO decreased the chlorophyll content seriously at GO concentrations of 0.8 and 2.0 mg mL^−1^ in hydroponic culture (Fig. 4a). It is well known that chlorophyll is the key component for photosynthesis, as chlorophyll molecules absorb light and transfer the energy to chemically reduce CO_2_. Consistently with the decreased chlorophyll content, the net photosynthetic rate decreased in hydroponic culture, which meant that the exposure to GO hindered the fixation of CO_2_ and this explained the weight loss (Fig. 2). In soil culture, the chlorophyll content only had a decrease at 0.2 mg mL^−1^ and there was no dose-dependent effect. The decrease of the net photosynthetic rate was observed at 2.0 mg mL^−1^. At 0.8 and 2.0 mg mL^−1^, the net photosynthetic rates in soil culture were statistically higher than those in hydroponic culture (Fig. 4b).

Similar trends were also observed in transpiration and stomatal conductance, which are vital for photosynthesis. GO showed a stimulating effect at 0.04 mg mL^−1^ and an inhibiting effect at 0.8 and 2.0 mg mL^−1^ in hydroponic culture for both transpiration and stomatal conductance, suggesting that the transportation of water and other substances might be affected by GO (Fig. 4c and d). In soil culture, the influence was only significant at 2.0 mg mL^−1^ and the values were larger than those in hydroponic culture. In addition, the intracellular CO_2_ levels stayed unchanged in both cultivation modes (Fig. S4†).

Many studies in the literature have concerned the photosynthesis of plants upon exposure to GO. Du *et al.* found that graphene had an adverse effect on chlorophyll synthesis in the green alga *Scenedesmus obliquus* and resulted in photosynthesis inhibition.^41^ Zhang *et al.* reported that 500 mg L^−1^ GO induced a 15% decrease of chlorophyll content, and the photosynthesis parameters including the maximum fluorescence yield (*F*_m_) and the maximum quantum efficiency of PSII photochemistry *F*_v_/*F*_m_ decreased.^36^ Separately, we observed the influence of GO on the chlorophyll content of the moss *Leucobryum glaucum*.^42^ At 2.0 mg mL^−1^, GO led to an increase of chlorophyll a and a decrease of chlorophyll b. The alteration of chlorophyll and photosynthesis by GO had significant environmental effects, which might disturb carbon fixation and agricultural production. Here, we showed that GO had a greater influence on photosynthesis in hydroponic cultivation. Therefore, GO might have more effects on aquatic plants, which require further investigation.

### Morphological changes

**Fig. 5:**
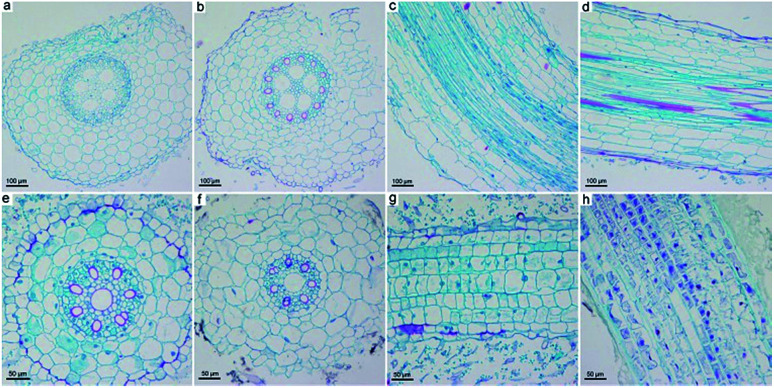
Optical microscopy investigation of the paraffin sections of naked oat roots in soil culture (a–d) and hydroponic culture (e–h). (a, c, e, g) Naked oats without GO exposure; (b, d, f, h) naked oats exposed to GO (2.0 mg mL^−1^).

Beyond the weight gain and photosynthesis, GO also influenced the structure and ultrastructure of naked oats. We focused on the root samples, because previous results indicated that GO mainly accumulated in the roots and induced toxicity there. As shown in Fig. 5a–d, the intact structure of the root samples in soil culture were observed in both the control and GO exposed groups. The epidermis, cortex, endodermis and vascular bundle could be clearly distinguished. However, in hydroponic culture, the root sample of the GO exposed group had structural changes, including a break of the epidermis, an irregular shape and changes to the vacuoles (Fig. 5e–h). The central axis of the root samples was kept intact even at a GO concentration of 2.0 mg mL^−1^, but shrinkage of the central axis was observed.

**Fig. 6:**
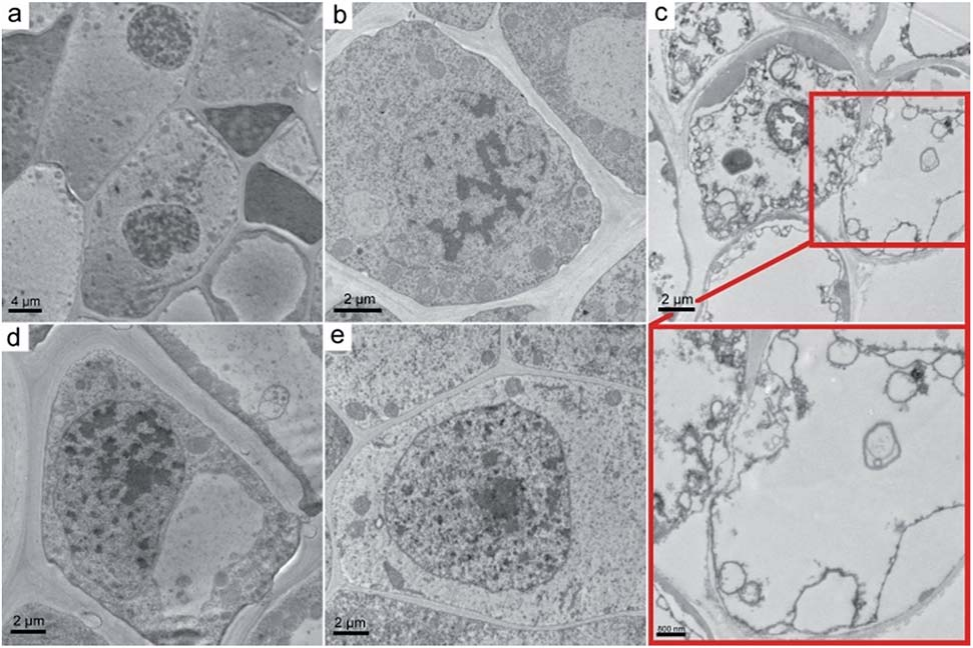
Representative TEM images of naked oat roots in hydroponic culture (a–c) and soil culture (d, e). (a, d) Naked oats without GO exposure; (b) naked oats exposed to GO (0.4 mg mL^−1^); (c, e) naked oats exposed to GO (2.0 mg mL^−1^).

The ultrastructure of the oat roots was further investigated using TEM to examine the damage to root cells. In the control sample, the cell membrane was tightly appressed to the cell wall (Fig. 6a and d). The nucleus had a distinct nuclear membrane and nucleolus. At the GO concentration of 0.4 mg mL^−1^, slight plasmolysis was present in the root tissue from hydroponic culture, but the organelles were still well preserved (Fig. 6b). Severe ultrastructural damage was observed at 2.0 mg mL^−1^ GO (Fig. 6c). The typical nucleus structure was lost and the organelles broke down. It was hard to distinguish the disrupted cellular components. Serious cell membrane and wall separation occurred. Overall, the ultrastructure observations supported the suggestion that GO induced more ultrastructural changes in hydroponic culture than in soil culture.

### Oxidative stress

Oxidative stress is a widely accepted mechanism of nanotoxicity. For plants, several studies have confirmed the oxidative damage that GO causes to roots and leaves. Here, we measured the glutathione (GSH)/malondialdehyde (MDA) and H_2_O_2_/catalase (CAT) levels to reflect the potential oxidative stress of root samples initiated by GO.

**Fig. 7:**
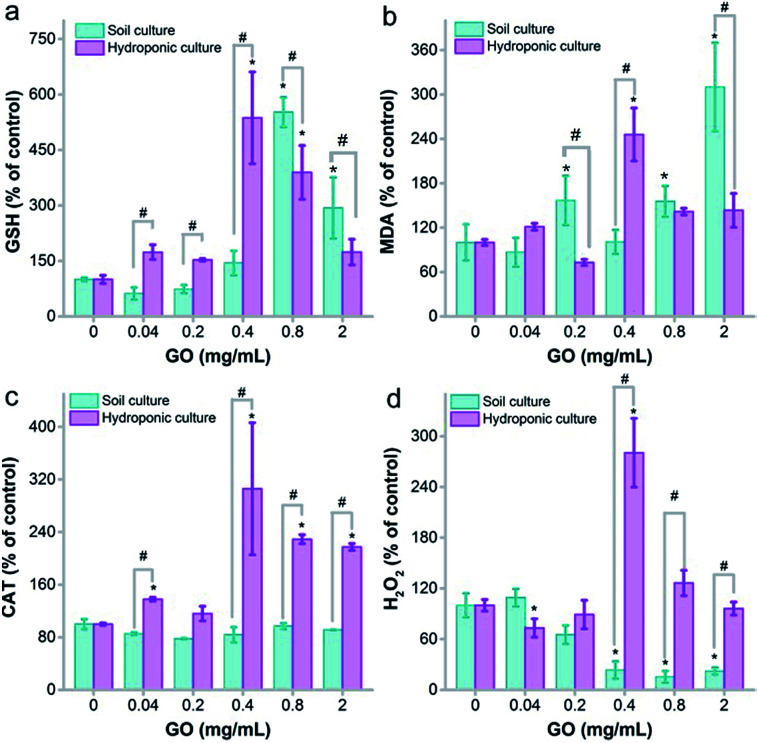
Oxidative stress of the naked oat roots exposed to GO in soil culture and hydroponic culture (*n* = 5). (a) GSH; (b) MDA; (c) CAT; (d) H_2_O_2_. * *p* < 0.05 compared to the control group; # *p* < 0.05 between soil and hydroponic culture.

The root tissues triggered oxidative stress to GO at 0.04 mg mL^−1^ and higher in the hydroponic environment. The GSH level showed increases from 0.04 mg mL^−1^ to 0.4 mg mL^−1^ in hydroponic culture, which could be regarded as a protective pathway of the root against oxidative damage. At 0.8 mg mL^−1^, the GSH level decreased, but the GSH level was still higher than that of the control at 2.0 mg mL^−1^ (Fig. 7a). However, the MDA levels increased at all GO concentrations, whereas MDA was supposed to decrease as the GSH levels increased (Fig. 7b). Similar trends were observed for soil culture, but the increases of GSH and MDA appeared later. CAT is the enzyme that decomposes intracellular H_2_O_2_. Naked oat roots up-regulated the CAT levels in hydroponic culture with exposure to GO, while the CAT levels stayed constant in soil culture (Fig. 7c). Just like MDA, H_2_O_2_ increased with the up-regulation of CAT levels in hydroponic culture. The H_2_O_2_ levels decreased in soil culture with exposure to GO (Fig. 7d). Overall, there was significant oxidative stress initiated by GO in both hydroponic and soil cultures. The oxidative stress was more serious in hydroponic culture and this could explain the toxicity of GO.

In the literature, there are a tremendous number of studies reporting oxidative stress as the toxicological mechanism caused by graphene in plants and other living creatures. Anjum *et al.* found that GO induced an increase of H_2_O_2_, ascorbate peroxidase and CAT levels.^24^ Zhang *et al.* reported an increase of MDA, reactive oxygen species (ROS), superoxide dismutase (SOD) and peroxidase (POD) levels in alga after exposure to reduced GO.^36^ Begum *et al.* assigned the toxicity of GO to oxidative damage in the leaves of cabbage, tomato and red spinach.^34^ Cheng *et al.* found that the MDA content did not increase significantly in *B. napus* L. roots exposed to GO.^35^ Wen *et al.* discovered that a low concentration of sulfonated graphene (SG) could scavenge ROS in roots and improve the health state of maize in response to hydrated graphene ribbon exposure.^43^ However, a high dose of SG promoted the generation of ROS and led to cell death in the roots. Together with our observations, we concluded that oxidative stress was the toxicological mechanism of GO in naked oats in both cultivation modes.

### Retention of GO on vermiculite

**Fig. 8:**
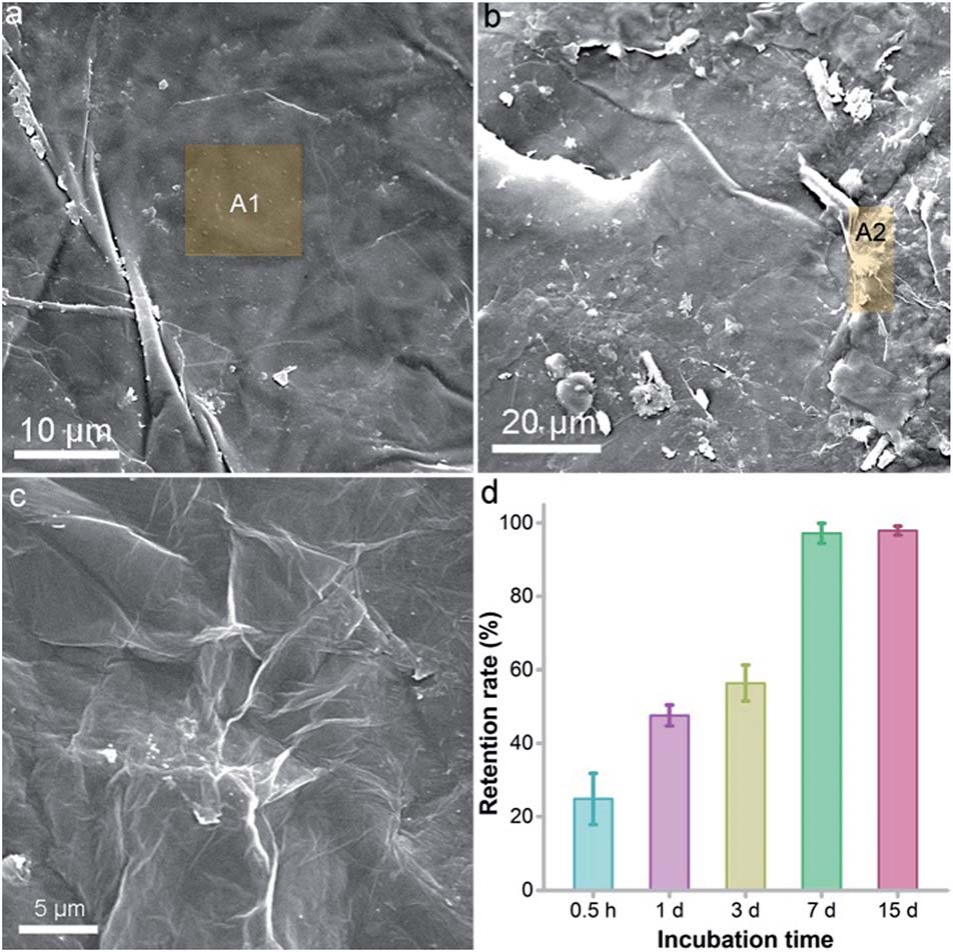
Retention of GO on vermiculite. (a) SEM of pure vermiculite; (b) SEM image of GO–vermiculite; (c) SEM image of pure GO; (d) retention rate of GO on vermiculite. The brown shaded areas (A1 and A2) were analysed using EDX for carbon identification.

According to the literature, GO had different transportation and retention behaviours in different media.^29–33^ Generally, the transportation of GO is harder in a solid medium than in a liquid medium. GO would hardly be available to the plant roots due to the strong binding to soil, thus the toxicity of GO might be alleviated. To verify our hypothesis, we investigated the morphology of a GO–vermiculite mixture under SEM and quantified the retention rate of GO on vermiculite. The surface of pure vermiculite was flat with some large wrinkles (Fig. 8a). The energy dispersive spectroscopy (EDS) result of the A1 area indicated the existence of O (38.6 wt%), Mg (9.7 wt%), Al (9.7 wt%), Si (21.4 wt%), K (5.22 wt%), Ca (1.1 wt%), Ti (1.1 wt%) and Fe (13.2 wt%), which is consistent with the chemical compositions of (Mg, Fe^2+^, Fe^3+^)_3_[(Si, Al)_4_O_10_](OH)_2_·4H_2_O. Ca, Ti and K were frequently detected elements in vermiculite, too. No C was found in vermiculite. After incubation with GO, there were small aggregates with tiny wrinkles on the vermiculite surface (Fig. 8b). The aggregates had a similar morphology to that of lyophilized GO (Fig. 8c). The EDS of the A2 area in GO–vermiculite showed a high C content (25.2 wt%), which was the direct identification of GO. This suggested that GO adsorbed onto the vermiculite surface after incubation.

To quantify the retention of GO on vermiculite, we incubated GO with vermiculite and washed the mixture in a column with water. As shown in Fig. 8d, a short incubation of 0.5 h resulted in a retention rate of 24.8%. With the elongation of incubation time, the retention of GO on vermiculite increased greatly. At 7 d, the retention rate reached 99.3% and stayed unchanged thereafter. Therefore, our results indicated that GO interacted strongly with vermiculite and was trapped there, losing its mobility. The restriction of GO on the vermiculite surface might weaken the contact of GO with naked oat roots and block the uptake of GO by roots. To verify this, we imaged the G band of GO on the surface of the roots after the removal of detached GO by mild shaking. As shown in Fig. S5,† the intensity of the G band was much stronger in the hydroponic culture group than in the soil culture group. The spectra of the most intense data points in both culture systems are listed, where a higher G band intensity was found in hydroponic culture. It can be concluded that fewer GO sheets were attached to the roots in soil culture, which is likely due to the graphene–soil interaction. Consequently, the toxicity of GO to naked oats showed a significant decrease in soil culture compared to that in hydroponic culture. This is a reminder that the transportation/mobility of GO in culture media must be carefully considered when evaluating its environmental toxicity.

## Conclusions

In conclusion, hydroponic culture exaggerated the toxicity of GO to naked oats, whereas the toxicity was largely alleviated in soil culture. GO induced growth inhibition, photosynthesis disturbance and morphological changes to naked oats in hydroponic culture. The toxicological mechanism was associated with oxidative stress. Due to the strong interaction between GO and vermiculite, the transportation of GO in vermiculite was limited and the contact between naked oat roots and GO was obviously reduced. Consequently, the toxicity of GO was significantly reduced in soil culture. Our results suggested that the cultivation method should be carefully considered when evaluating the toxicity of nanomaterials to plants. Hydroponic culture could reveal the potential hazards more sensitively, but the situation could be milder in soil culture. Therefore, the practical hazards of nanomaterials to the environment might be lower than concluded from hydroponic evaluation.

## Experimental

### Preparation and characterization of GO

Graphite powder (purity of 99.85%) was purchased from Shanghai Huayi Group Co., China. Graphite was oxidized following the modified Hummers’ method, as described in our previous reports.^19,22^ The as-prepared GO was characterized using TEM (JEM-200CX, JEOL, Japan), AFM (SPM-9600, Shimadzu, Japan), XPS (Axis Ultra, Kratos, UK), Raman spectroscopy (inVia, Renishaw, UK) and IR spectroscopy (Avatar 370, Thermo Nicolet, USA).

### Transportation of GO in vermiculite

To evaluate the transportation of GO in vermiculite, 100 mL vermiculite was added to 27 mL GO dispersion (7.4 mg mL^−1^), 10 mL 10-fold nutrient solution and 18 mL water. At 30 min, 1 d, 3 d, 7 d and 15 d post-mixing, 10 mL of the GO–vermiculite mixture was placed into a glass column (1.5 cm in diameter) and washed with water. The effluent was collected until the colour of the effluent vanished. The GO concentration of the effluent was determined by measuring the absorbance at 400 nm and the retention rate of GO was calculated.

### Plant cultivation and GO exposure

Naked oat (*A. sativa* L.) seeds were obtained from Taigu County Lvbao Seed Industry Co., China. Vermiculite (diameters of 1–3 mm) was purchased from North Mining Processing Co., China. Hoagland nutrient solution was used for the plant cultivation. The constitution of the modified Hoagland solution was listed in our previous report.^22^

Naked oat seeds were soaked in 15% NaCl for 30 min, followed by being soaked twice in deionized water for 15 min each before germination. Groups of 30 seeds were each placed on a piece of filter paper in a Petri dish (diameter of 9 cm). Then, 10 mL Hoagland nutrient solution was added to the Petri dish. The seeds were incubated in a dark incubator at a humidity of 60% and 20 °C for 4 d. For cultivation in the hydroponic system, the germinated seeds (root length of 3–5 cm and germ length of 1–3 cm) were transferred to 100 mL beakers containing 0–2.0 mg mL^−1^ GO. The cultivation parameters were set as: day/night cycle of 12 h/12 h, 24 000 lx for the day cycle, a humidity of 60%, and a temperature of 23/18 °C. During the observation period, Hoagland nutrient solution was added daily to maintain the volume of the hydroponic system at 100 mL. For cultivation in vermiculite, the germinated seeds were placed in beakers containing 100 mL of vermiculite supplemented with 55 mL of Hoagland nutrient solution and different concentrations of GO (0–2.0 mg mL^−1^). Hoagland nutrient solution was added daily in amounts of 5 mL (1–5 d), 10 mL (6–10 d) or 15 mL (11–15 d). The naked oat seedlings were harvested from both cultivation systems for toxicity evaluation at day 15 post-planting.

### Toxicity evaluations

After harvesting, the root samples and aboveground parts were carefully separated, washed, had the attached water removed with filter paper, and weighed to obtain the fresh weights. After drying for 12 h at 90 °C in an oven, the samples were weighed again to obtain the dry weights.

Another set of seedlings were subjected to the chlorophyll measurements using a chlorophyll meter (SPAD-502plus, Konica Minolta Co., Japan). For the chlorophyll measurements, each seedling was measured at five independent sites. The net photosynthetic rate, stomatal conductance, transpiration rate, and intercellular CO_2_ concentration were measured using a portable photoactivator (Yaxin-1102, Beijing YaXin Liyi Technology Co., China). Then, the lengths of the roots, stems and leaves were measured.

For oxidative stress assays, plant samples (0.5 g of each) were collected and kept at −20 °C before homogenization in ice-cold saline (4.5 mL). The homogenates were centrifuged at 4000 rpm for 10 min to obtain the supernatant. Coomassie brilliant blue was used to determine the supernatant protein concentration as described in our previous report. All kits for oxidative stress were bought from Nanjing Jiancheng Bioengineering Institute, China. The MDA, H_2_O_2_, GSH and CAT levels were analysed strictly following the kit instructions on a UV-vis spectrometer (UV-1800, Mapada Co., China).

For microscopy, the fresh root samples were cut into pieces 0.5–1 cm in length and fixed with formaldehyde–acetate–alcohol solution for the paraffin section. The standard protocol was applied and the sections were stained using safranine and fast green. The images of the root paraffin sections were taken under an optical microscope (CAB-30PC, Cabontek Co., Chengdu, China). For TEM observations, the roots samples were fixed using 2.5% glutaraldehyde, post-fixed in 1% osmium tetroxide, dehydrated in a graded alcohol series, embedded in epoxy resin, cut with an ultramicrotome, post-stained with uranyl acetate and lead citrate, and finally placed on the copper meshes for TEM investigation.

### Statistical analysis

All data are presented as the mean of five individual observations with the standard deviation (mean ± SD). The significance was calculated using a one-way ANOVA test and two-way ANOVA test (Table S1†) using IBM SPSS19.0 software. The difference was considered significant if *p* < 0.05. The *post hoc* test was performed when *p* was smaller than 0.05.

## Conflicts of interest

There are no conflicts to declare.

## Supplementary Material

RA-008-C8RA01753K-s001
