# Supplementary material for: Toxicity of graphene oxide to naked oats (Avena sativa L.) in hydroponic and soil cultures
Source: RSC Adv. 2018 Apr 24;8(28):15336–43. doi: 10.1039/c8ra01753k (PMC9080036; doi:10.1039/c8ra01753k)
Supplement: RA-008-C8RA01753K-s001 [file RA-008-C8RA01753K-s001.pdf]

Electronic Supplementary Material (ESI) for RSC Advances.
This journal is © The Royal Society of Chemistry 2018

Electronic supplementary information

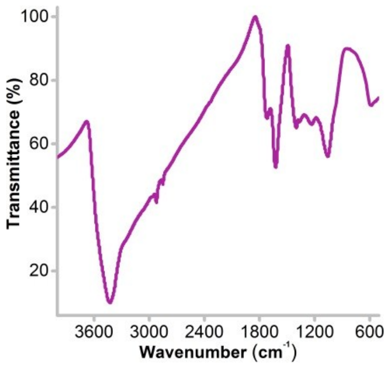

Fig. S1 IR spectrum of GO.

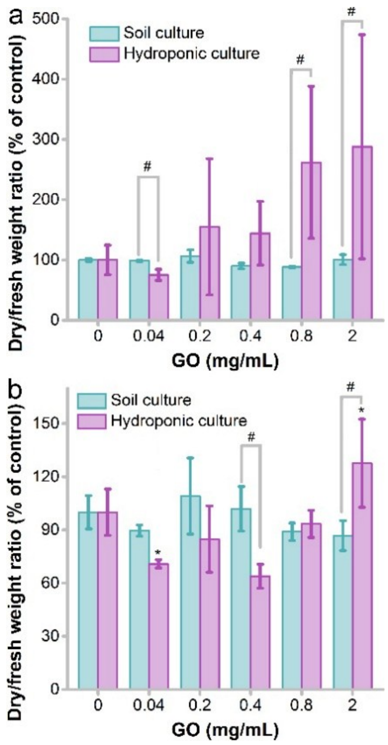

Fig. S2 Dry/fresh weight ratio of naked oats exposed to GO in soil culture and hydroponic culture (n=5). (a) root samples; (b) aboveground parts. * $p < 0.05$ comparing to the control group; # $p < 0.05$ between soil and hydroponic culture.

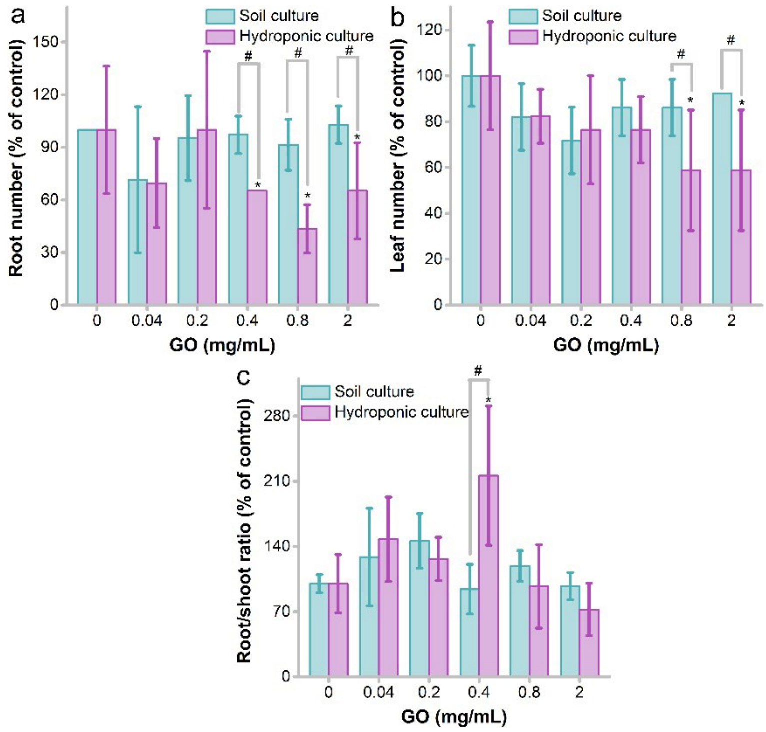

Fig. S3 (a) root number, (b) leaf number and (c) root/shoot ratio of naked oats exposed to GO in soil culture and hydroponic culture (n=5). * $p < 0.05$ comparing to the control group; # $p < 0.05$ between soil and hydroponic culture.

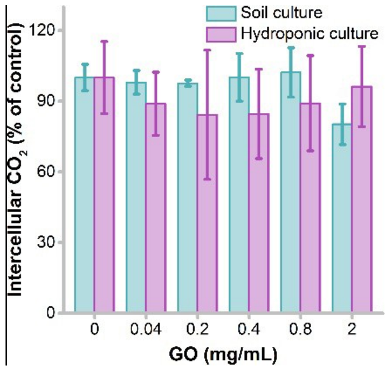

Fig. S4 Intracellular CO_2 level of naked oats exposed to GO in soil culture and hydroponic culture (n=5).

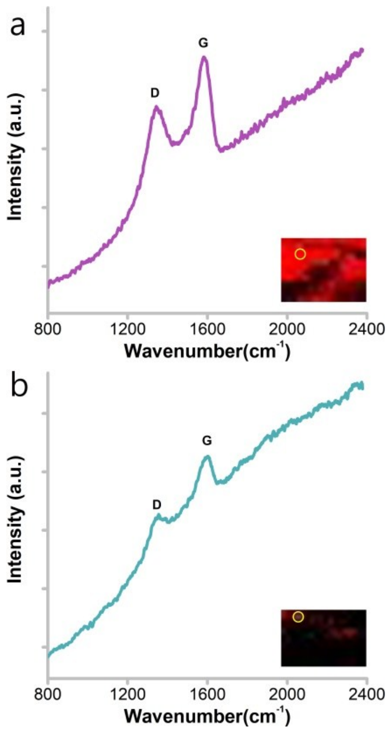

Fig. S5 Raman spectra of GO attached on the roots of naked oat. (a) hydroponic culture; (b) soil culture. The insects are the 2D mapping of G band intensity on the root surface (60μm × 40μm). The spectra were collected from the datum points of highest intensity as indicated by the circles.

Table S1 Statistical results of Two-way ANOVA for "GO concentration" and "Cultivation mode". * $p < 0.05$.

Source	Measurement data source	Type IIIsum of squares	df	Mean square	F	Sig
GO concentration	Root number	0.647	5	0.129	2.035	0.093
	Aboveground part length	1.688	5	0.338	3.797	0.006*
	Root/shoot ratio	3.407	5	0.681	3.807	0.006*
	Chlorophyll	0.856	5	0.171	3.497	0.010*
	Root length	3.675	5	0.735	4.564	0.002*
	Leaf number	0.459	5	0.092	2.235	0.068
	Fresh weight of aboveground parts	2.647	5	0.529	6.405	0.001*
	Dry weight of aboveground parts	1.700	5	0.340	5.029	0.003*
	Dry/fresh weight ratio of aboveground parts	0.319	5	0.064	2.381	0.070
	Fresh weight of root	2.744	5	0.549	5.114	0.003*
	Dry weight of root	1.721	5	0.344	3.503	0.017*
	Dry/fresh weight ratio of root	5.172	5	1.034	1.186	0.346
	Net photosynthetic rate	3.933	5	0.787	14.816	0.000*
	Transpiration	4.209	5	0.842	8.716	0.000*
	Stomatal conductance	5.294	5	1.059	7.179	0.000*
	Intracellular CO_2 level	0.160	5	0.032	0.468	0.798
	GSH	57.151	5	11.430	22.847	0.000*
	MDA	2.700	5	0.540	7.957	0.001*
	CAT	2.683	5	0.537	2.621	0.068
	H_2O_2	2.262	5	0.452	9.149	0.000*
Cultivation mode	Root number	0.705	1	0.705	11.085	0.002*
	Aboveground part length	0.106	1	0.106	1.191	0.281
	Root/shoot ratio	0.204	1	0.204	1.142	0.291
	Chlorophyll	0.555	1	0.555	11.342	0.002*
	Root length	0.006	1	0.006	0.040	0.843
	Leaf number	0.158	1	0.158	3.845	0.056
	Fresh weight of aboveground parts	0.072	1	0.072	0.869	0.361
	Dry weight of aboveground parts	0.009	1	0.009	0.137	0.715
	Dry/fresh weight ratio of aboveground parts	0.031	1	0.031	1.152	0.294
	Fresh weight of root	0.070	1	0.070	0.656	0.426

	Dry weight of root	0.105	1	0.105	1.067	0.312
	Dry/fresh weight ratio of root	4.661	1	4.661	5.345	0.030*
	Net photosynthetic rate	0.918	1	0.918	17.286	0.000*
	Transpiration	1.010	1	1.010	10.457	0.002*
	Stomatal conductance	0.602	1	0.602	4.083	0.049*
	Intracellular CO_2 level	0.061	1	0.061	0.889	0.351
	GSH	1.248	1	1.248	2.494	0.135
	MDA	0.106	1	0.106	1.559	0.231
	CAT	6.204	1	6.204	30.305	0.000*
	H_2O_2	3.438	1	3.438	69.530	0.000*
GO concentration * Cultivation mode	Root number	0.489	5	0.098	1.536	0.199
	Aboveground part length	0.843	5	0.169	1.896	0.115
	Root/shoot ratio	3.868	5	0.774	4.323	0.003*
	Chlorophyll	0.879	5	0.176	3.590	0.008*
	Root length	3.555	5	0.711	4.414	0.002*
	Leaf number	.288	5	0.058	1.400	0.243
	Fresh weight of aboveground parts	3.017	5	0.603	7.300	0.000*
	Dry weight of aboveground parts	1.155	5	0.231	3.416	0.019*
	Dry/fresh weight ratio of aboveground parts	0.576	5	0.115	4.295	0.007*
	Fresh weight of root	1.752	5	0.350	3.265	0.023*
	Dry weight of root	0.992	5	0.198	2.018	0.114
	Dry/fresh weight ratio of root	5.509	5	1.102	1.264	0.313
	Net photosynthetic rate	1.583	5	0.317	5.962	0.000*
	Transpiration	1.227	5	0.245	2.541	0.041*
	Stomatal conductance	1.861	5	0.372	2.524	0.043*
	Intracellular CO_2 level	0.095	5	0.019	0.278	0.923
	GSH	22.371	4	5.593	11.179	0.000*
	MDA	3.770	4	0.943	13.887	0.000*
	CAT	3.645	4	0.911	4.452	0.014*
	H_2O_2	6.043	4	1.511	30.553	0.000*
